# Mechanisms regulating the sorting of soluble lysosomal proteins

**DOI:** 10.1042/BSR20211856

**Published:** 2022-05-13

**Authors:** İçten Meraş, Juliette Maes, Stephane Lefrancois

**Affiliations:** 1 Centre Armand-Frappier Santé Biotechnologie, Institut national de la recherche scientifique, Laval, Canada; 2 Department of Anatomy and Cell Biology, McGill University, Montreal, Canada H3A 0C7; 3 Centre d’Excellence en Recherche sur les Maladies Orphelines - Fondation Courtois (CERMO-FC), Université du Québec à Montréal (UQAM), Montréal, Canada H2X 3Y7

**Keywords:** endoplasmic reticulum, endosomes, intracellular transport, Lysosomes, neurodegeneration, neuronal ceroid lipofuscinosis

## Abstract

Lysosomes are key regulators of many fundamental cellular processes such as metabolism, autophagy, immune response, cell signalling and plasma membrane repair. These highly dynamic organelles are composed of various membrane and soluble proteins, which are essential for their proper functioning. The soluble proteins include numerous proteases, glycosidases and other hydrolases, along with activators, required for catabolism. The correct sorting of soluble lysosomal proteins is crucial to ensure the proper functioning of lysosomes and is achieved through the coordinated effort of many sorting receptors, resident ER and Golgi proteins, and several cytosolic components. Mutations in a number of proteins involved in sorting soluble proteins to lysosomes result in human disease. These can range from rare diseases such as lysosome storage disorders, to more prevalent ones, such as Alzheimer’s disease, Parkinson’s disease and others, including rare neurodegenerative diseases that affect children. In this review, we discuss the mechanisms that regulate the sorting of soluble proteins to lysosomes and highlight the effects of mutations in this pathway that cause human disease. More precisely, we will review the route taken by soluble lysosomal proteins from their translation into the ER, their maturation along the Golgi apparatus, and sorting at the trans-Golgi network. We will also highlight the effects of mutations in this pathway that cause human disease.

## Introduction

First discovered by Christian de Duve, lysosomes perform several key cellular functions, and their dysfunction has been implicated in several human pathologies (reviewed in [1,2]). One of the central functions of this organelle is to degrade cellular materials, originating from autophagy, phagocytosis or endocytosis. Substrates to be degraded include proteins, carbohydrates, nucleic acids as well as lipids, and is achieved by more than 70 soluble enzymes, along with enzyme activators and protective factors present in the lumen. Lysosomes are highly dynamic organelles in constant flux which fuse and reform following processes such as autophagosome/lysosome fusion [3]. The lysosomal membrane contains several types of membrane proteins that have a wide variety of functions including the protection of the membrane, the control of pH and ion homeostasis through membrane permeable channels and pumps. Furthermore, these membrane proteins also participate in the formation of membrane contact sites or the fusion of lysosomes with other organelles (reviewed in [4]). In this review, we provide an up-to-date view of the mechanisms by which soluble lysosomal proteins are sorted through various cellular compartments, starting at the endoplasmic reticulum (ER) all the way to their final destination in lysosomes. We will also emphasize the interdependence of anterograde and retrograde trafficking and their consequences on lysosomal activity. In the process, mutations in certain pathways will be discussed, when these changes result in human pathology.

## Sorting through the endoplasmic reticulum

Most soluble lysosomal proteins begin their journey as peptides that contain a 20–25 amino acid signal sequence that enables their translocation from the cytosol into the lumen of the ER during translation [5]. Some exceptions to this conventional route have been identified. For example, ceroid lipofuscinosis neuronal 5 (CLN5) and phospholipase D3 are initially translated as type II integral membrane proteins and are subsequently cleaved to produce mature soluble lysosomal proteins [6,7]. In the case of CLN5, it is cleaved by members of the signal peptide peptidase-like proteases (SPPL) family of intramembrane proteases, which are localized at the ER/Golgi interface [6]. On the other hand, cleavage of phospholipase D3 occurs in an acidic compartment, with a yet identified mechanism [7].

Newly synthesized proteins containing the 20–25 amino acid signal peptide undergo their first modifications: a signal peptide peptidase cleaves the signal peptide, while N-glycosylations are added to asparagine residues of Asn-X-Ser/Thr motifs by the oligosaccharyl transferase (OST) [8]. These soluble proteins move through the ER, are packaged into COPII vesicles and move on to the Golgi apparatus [9]. The efficiency and selectivity of protein sorting depends on specific interactions and cooperation between soluble cargos, cargo receptors and coat proteins. Transport out of the ER is mediated by the COPII coatomer complex [10]. This coat complex is recruited to the ER by the small GTPase Sar1 [11]. When activated and membrane bound, Sar1 acts as a docking factor for the Sec23 and Sec24 components of COPII to form a pre-budding complex at ER exit sites (ERES) (Figure 2). Sec24 is responsible for cargo binding and concentrating proteins into forming vesicles [12]. Next, the Sec13 and Sec31 subunits are recruited and function to deform the membrane to produce nascent vesicles, which are subsequently cleaved from the ER membrane [12] (Figure 2). Sec24 recognizes various sorting signals such as the phenylalanine-phenyalanine (Phe-Phe) motif present in the cytosolic C-terminal tail of the cargo receptor LMAN1/ERGIC53, which is responsible for the sorting of glycosylated proteins such as the lysosomal enzyme cathepsin C, and the well characterized ΦXΦXΦ (Φ is a hydrophobic amino acid, and X is any amino acid) ER export motif [13,14]. Although some ER cargo receptors have been identified, the mechanism regulating the sorting of soluble lysosomal proteins at the ER–Golgi interface remains only partially understood.

The mechanism as to how soluble lysosomal proteins move through the ER and are exported to the Golgi apparatus was recently demonstrated. Ceroid lipofuscinosis neuronal 6 (CLN6) and CLN8 are ubiquitously expressed and localized at the ER [15–19]. Mutations in either of the genes result in a neurodegenerative disease known as neuronal ceroid lipofuscinosis (NCL) [20,21]. Beyond CLN6 and CLN8, mutations in a total of 13 different genes (*CLN1–CLN8* and *CLN10–CLN14*), are the cause of NCL. Disease onset can occur at any stage in life, but most often affects children. Common symptoms include cognitive regression, seizures, visual failure, and ataxia. At a cellular level, defective lysosomal function is a hallmark of these diseases, resulting in the accumulation of ceroid lipofuscin, among other molecules [22].

Using an elegant screening method based on interactions in live cell using bimolecular fluorescence complementation (BiFC), it was shown that CLN8 can interact with several soluble lysosomal proteins including other NCL proteins: PPT1 (CLN1), TPP1 (CLN2), cathepsin D (CLN10) and cathepsin F (CLN13) [23]. To test for a functional relationship, the localization of cathepsin D was compared in cortical and cerebellar sections from CLN8-deficient mice to wild-type mice, while the localization of TPP1 was compared in patient-derived CLN8-deficient fibroblasts with cells obtained from healthy donor. A significant decrease in the amount of cathepsin D was found in the LAMP1-positive compartment (endolysosomal compartment) of CLN8-deficient cells compared to wild-type cells, while the same was true for TPP1 in patient-derived cells compared to healthy cells [23]. Finally, a proteomic analysis of purified lysosomes found a significant decrease in a number of soluble lysosomal proteins, but little change in lysosomal integral membrane proteins in CLN8-deficient cells, supporting a role for this protein in trafficking soluble lysosomal proteins Table 1.

**Table 1:** Diseases associated with genes participating in the trafficking of soluble lysosomal proteins

Protein name	Function	Cellular localization	Disease	References
**AP-1** Heterotetrameric Adaptor Protein Complex	Interacts with lysosomal sorting receptors and recruits clathrin coat to TGN membrane	TGN	MEDNIK syndrome MEDNIK-like Syndrome X-linked Intellectual Disability Pettigrew Syndrome Pustular Psoriasis	[104–112]
**CLN3** Ceroid Lipofuscinosis Neuronal 3	Rab7A/retromer interaction Rab7A/sortilin interaction	Endolysosomes	Neuronal Ceroid Lipofuscinosis	[147]
**CLN5** Ceroid Lipofuscinosis Neuronal 5	Binds to CLN3 and and modulates its activity	Endolysosomes	Neuronal Ceroid Lipofuscinosis	[148]
**CLN6** Ceroid Lipofuscinosis Neuronal 6	Lysosomal soluble protein trafficking from ER- to-Golgi function together with CLN8	ER	Neuronal Ceroid Lipofuscinosis	[22,23]
**CLN8** Ceroid Lipofuscinosis Neuronal 8	Lysosomal soluble protein trafficking from ER-to-Golgi, functions together with CLN6	ER	Neuronal Ceroid Lipofuscinosis	[22,23]
**LIMP2**	Receptor protein for β-Glucocerebrosidase	Endolysosomes	Parkinson's disease Gaucher disease Progressive Myoclonic epilepsy	[81]
**N-acetylglucosiamne-1- phosphoptransferase**	Catalyzes Man-6-P modification on lysosomal enzymes as their recognition marker for their proper lysosomal transport	Golgi	Mucolipidosis II	[44,45]
**Rab7A**	Small GTPase: acts as a molecular switch for early-late endosome maturation, recruitment and stabilization of retromer complex	Endolysosomes	Charcot–Marie–Tooth Disease	[156]
**Retromer** Trimeric protein complex VPS26/29/35	Protein complex that interacts with cargo receptors and recycles them back to TGN	Endolysosomes	Parkinson’s disease Alzheimer’s disease Amyotrophic lateral sclerosis (ALS) Neuronal Ceroid Lipofuscinosis	[147–155]

CLN6 is the only other NCL related protein located in the ER. CLN6 interacts with a number of soluble lysosomal proteins, and its depletion results in the significant decrease of soluble lysosomal proteins localizing to lysosomes [24]. This decrease in localization also corresponds to diminished lysosomal protein activity. Soluble enzyme depletion in the lysosomal compartment was also observed in CLN6-deficient mice, suggesting that both CLN6 and CLN8 are involved in lysosomal soluble proteins trafficking [24]. Their role was recently identified as a two-step process.

**Figure 1:**
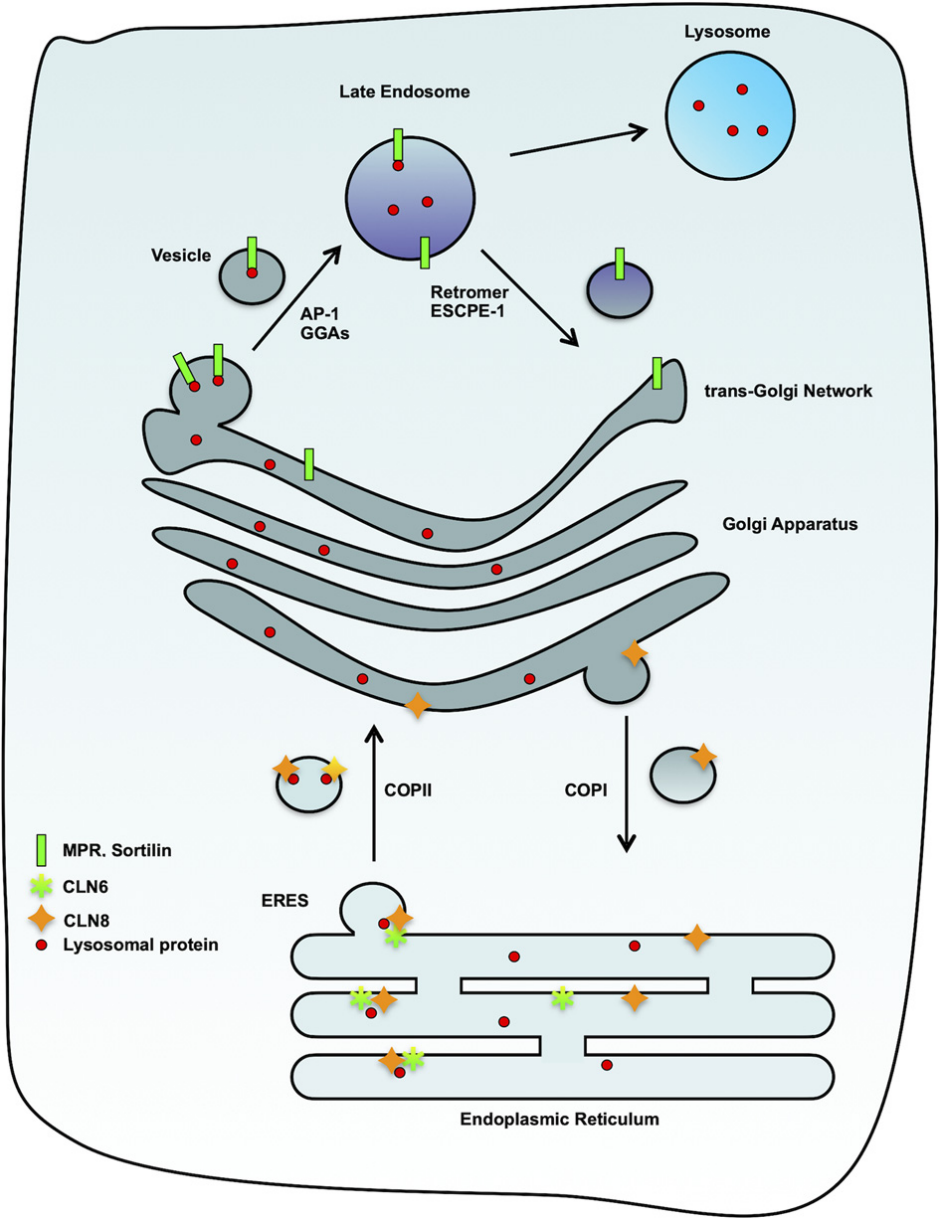
Schematic representation of sorting pathways taken by lysosomal cargo proteins

**Figure 2:**
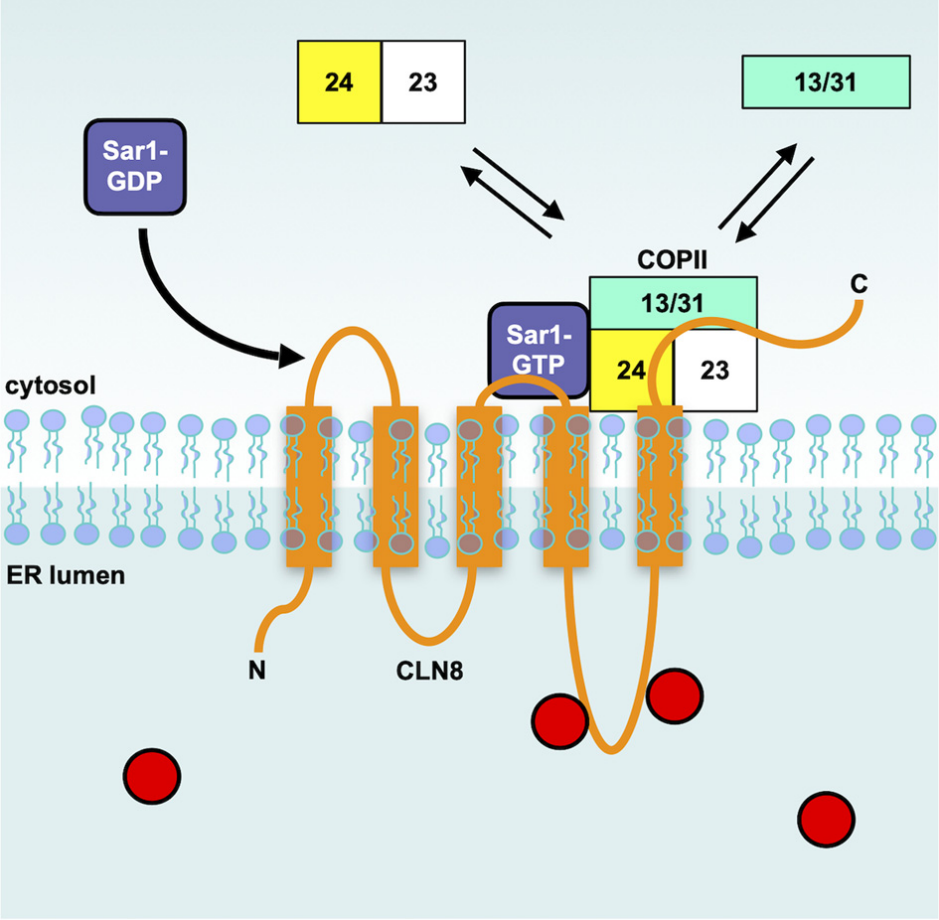
CLN8 sorting at the ER Soluble cargo (red circles) are recognized by the luminal loop of CLN8 (orange) in the ER. Activated Sar1 is recruited to the membrane, recruits COPII, and packages CLN8 bound to cargo into trafficking vesicles.

First, CLN8 forms a homodimer that interacts with CLN6 on the membrane of the ERES to create a complex named EGRESS (ER-to-Golgi relaying of enzymes of the lysosomal system). This complex is responsible for the recruitment of soluble lysosomal proteins to promote their transport to the Golgi apparatus via the large luminal loop of CLN6 and the second luminal loop of CLN8 (Figure 2). Then, CLN8 interacts with the Sec24 subunit of COPII, which packages the receptor along with cargo into budding vesicles [23]. Sec24 recognizes the specific export signal VDWNF (ΦXΦXΦ) present at the cytosolic tail of CLN8 [23]. COPII coated vesicles, loaded with cargo are exported from the ER and transported to the cis-cisternae of the Golgi apparatus, while CLN6 remains in the ER [24] (Figures 1 and 2).

## The Golgi apparatus: a trafficking hub

The Golgi apparatus is the cellular centre for the modification, sorting and trafficking of cargo proteins and lipids to their final destinations. Newly synthesized proteins and lipids enter the cis-cisternae of the Golgi apparatus from the ER. While they progress through the Golgi, escaped ER resident proteins, as well as empty receptors are retrieved back to the ER, enabling the latter to take part in other cycles of trafficking. Once cargo proteins, such as soluble lysosomal proteins, and lipids reach the trans-cisternae and the trans-Golgi Network (TGN), which is the tubular structure of the most trans site of the Golgi complex, they leave for their final destinations [25–28]. How proteins are transported through the Golgi cisternae is still an active area of research. Several models have been proposed including (1) vesicular transport, (2) diffusion, (3) kiss and run, and (4) cisternae maturation.

(1) The vesicular transport model, as the name suggests, includes the formation of vesicles, in which cargos are packed and transported from one cisternae to another for further modifications by the enzymes within that cisternae and eventually exit from the Golgi. (2) In the diffusion model, small soluble cargo, such as albumin, transverse through the Golgi via interconnected Golgi stacks by simple diffusion [29]. However, this model excludes the transport of large cargo and probably underlies the co-existence of more than one model for intra-Golgi transport for different cargos. (3) In the symmetric kiss and run model, two Golgi compartments can fuse together through a transient narrow neck, which then undergoes fission to separate into two compartments. In the asymmetric kiss and run model, cargo molecules accumulate in a domain within the cisternae. The domain goes through fusion with the ‘accepting’ cisterna and subsequently undergoes fission with its originating cisterna [30]. The kiss and run model provides an advantage for cargo to move bidirectionally through the fusion neck without a separate mechanism. (4) Lastly, the Cisternal maturation model predicts that each cisternae moves and matures to distal cisternae. The key difference is that while the cargo stays in the cisternae, it is the Golgi resident proteins that recycle back to the previous cisternae. Each model poses its own advantages and disadvantages which are reviewed in detail here [31].

## Protein retrieval at the ER–Golgi interface

**Figure 3:**
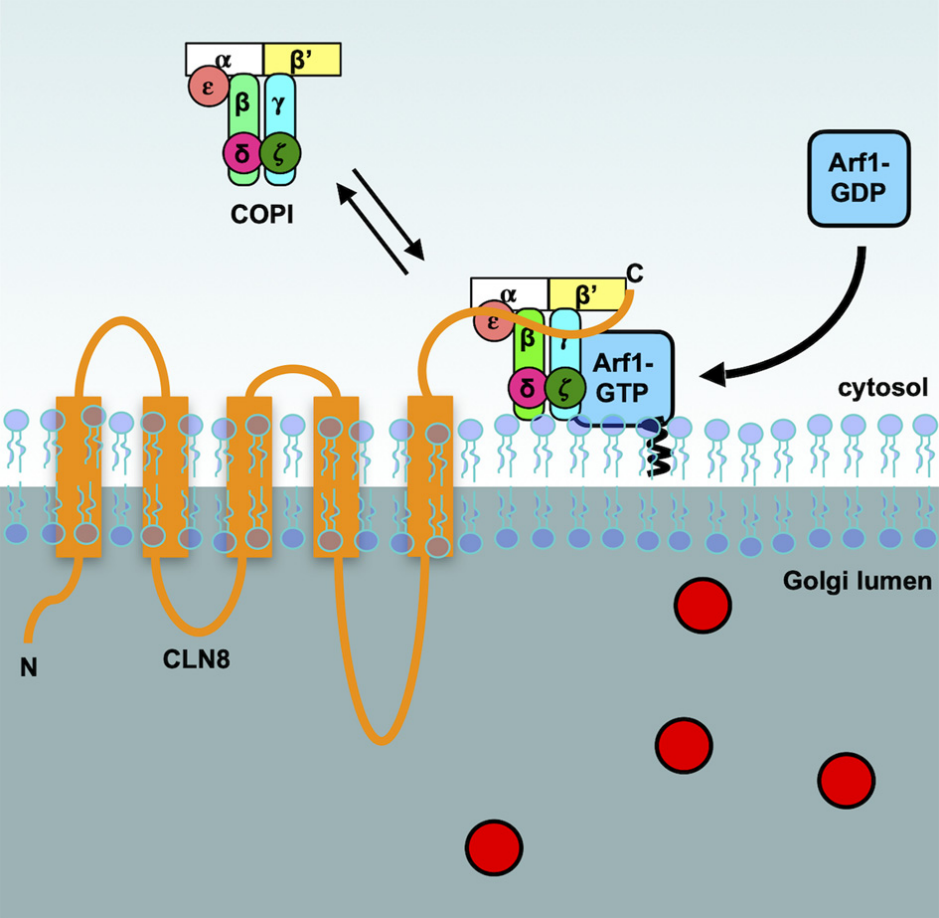
Retrieval of CLN8 from the Golgi Once CLN8 (orange) delivers cargo (red circles) to the Golgi apparatus, Arf1 recruits COPI to retrieve CLN8 back to the ER

At the cis-Golgi, empty receptors, mis-sorted or mis-folded proteins, along with escaped ER resident proteins are retrieved back to the ER. This retrograde transport is enabled by a network of vesicles trafficking from the cis-Golgi to the ER, a process mediated by the COPI complex (Figure 3). COPI is located in the cytosol and composed of seven subunits : α, β′, ε, β, δ, γ and ζ. α, β′ and ε subunits form a cage-like B-subcomplex whereas β, δ, γ and ζ subunits form an adaptor-like F-subcomplex. The formation of vesicles starts with the activation of the small GTPase, ADP-ribosylation Factor 1 (Arf1) by guanine nucleotide exchange factors (GEFs) such as GBF1 at the cis-Golgi [32]. Arf proteins undergo N-terminal myristoylation, which is an important step in anchoring Arf GTPases to membranes [33].

Activated, GTP-loaded Arf1 localizes to membranes of the cis-Golgi, interacting with the γζ and βδ subunits, leading to the recruitment of COPI [32,34]. Studies have shown that other Arf GTPases could also be involved in COPI recruitment [35]. ArfGEFs and Arf GTPase-activating proteins (ArfGAPs) such as ArfGAP1, 2 and 3, which return Arf1 to an inactive GDP bound form, were also shown to play important roles in promoting COPI recruitment, stability and vesicle budding (reviewed by [36]). Integral membrane proteins to be retrieved are then packaged into the nascent vesicle by interacting with COPI via specific sorting signals, while soluble proteins located in the lumen must bind to transmembrane receptors. Several retrieval signals and their receptors have been characterized (reviewed in [37]).

One of the most studied cargo receptors for ER-to-Golgi trafficking is the KDEL receptor (KDELR). This seven transmembrane domain containing protein localizes mainly to the cis-cisternae of the Golgi and is predicted to behave like a G-protein coupled receptor (GPCR) [38]. KDELR recognizes the KDEL (Lys-Asp-Glu-Leu) motif found on ER resident proteins and retrieves them back to the ER in a pH-dependent manner [39,40]. It was recently proposed that three amino acids of the signal binding pocket of KDELR (Y158, E127 and H12) could play an essential role in KDELR activation and cargo locking. Using quantum mechanical modelling, it is predicted that in the more acidic environment of lumen of the Golgi, H12 undergoes protonation, which would strengthen the hydrogen bond between Y158 and E127, resulting in a stronger interaction of KDEL-tagged proteins with the KDELR. Cargo binding then induces a conformational change of the KDELR and its activation, required for the recruitment of COPI complex [40,41]. KDELR activation also initiates a signaling cascade inside the Golgi complex that results in the phosphorylation of Src and Src Family Kinases (SFK). This signalling cascade regulates Golgi-to-ER trafficking and Intra-Golgi transport [38], such that SFK inactivation by inhibitors resulted in the accumulation of vesicular stomatitis virus G protein (VSVG) in the Golgi and prevented it from reaching the TGN [42]. While KDELR induced SFK activation regulates intra-Golgi trafficking, a PKA signaling cascade plays an important role in regulating Golgi-to-ER retrograde transport, as well as promoting the expression of genes involved in vesicular trafficking through CREB1 activation [38,42,43]. A recent study suggests that activation by KDELR signaling inside the Golgi also results in lysosomal repositioning, bringing lysosomes close to the perinuclear space, and sustains Golgi secretion [44].

## Protein maturation along the Golgi cisternae

Enzymes present in the different cisternae of the Golgi apparatus catalyze important post-translational modifications including phosphorylation, glycosylation, proteolytic cleavage and sulfation [45]. Some modifications are required for the proper sorting of cargos to their final destination, as well as their function. Indeed, in 1972, Hickman & Neufeld showed that soluble lysosomal enzymes required certain modifications to be sorted to lysosomes using skin-derived fibroblasts from I-cell disease patients [46]. This disease, also known as mucolipidosis II, is a rare lysosomal storage disorder caused by mutations in N-acetylglucosamine-1-phosphotransferase [45,46]. N-acetylglucosaminyl-1-phosphotransferase (GlcNac-1-phosphotransferase) and N-acetylglucosamine-1-phosphodiester α-N-acetylglucosaminidase act sequentially to synthesize mannose 6-phosphate (M6P) on many lysosomal soluble N-glycosylated enzymes. The latter enzyme is also known as the uncovering enzyme that exposes the M6P. N-acetylglucosamine-1-phosphodiester α-N-acetylglucosaminidase mainly localizes to the TGN, while a small amount can be found at the plasma membrane, suggesting the enzymes cycles between the TGN and the plasma membrane [45,46]. As such, tagging proteins with M6P separates proteins from a general secretion pathway, and targets them for sorting towards the lysosome.

## Cargo sorting in the TGN and beyond

The trans-Golgi Network (TGN), is the trans-most distal cisternae site of the Golgi apparatus and has a tubular network shape. TGN structure and size vary from one cell type to another, and incoming and outgoing trafficking to the TGN dynamically regulates its size and morphology [47–50]. In the classical point of view, the TGN is depicted as the ultimate sorting hub for cargo proteins and lipids, where they are sorted to their final destinations [47,51]. This view has been challenged with results from polarized Madin–Darby canine kidney (MDCK) cells implicated in biosynthetic cargo trafficking. Studies showed that, after cargos exit from the TGN, they visit endosomal compartments inside the cell before their final destinations, for example, common recycling endosomes, apical and basal sorting endosomes [51,52].

Whether sorting happens ultimately in the TGN or in post-TGN endosomal compartments, sorting of cargo proteins requires an elaborate collaboration between receptor, adaptor and coat proteins, which will be discussed below.

## The lysosomal sorting receptors

### The mannose 6-phosphate receptors

Newly tagged proteins with M6P are recognized by two different lysosomal sorting receptors, namely: the 46 kDa cation-dependent (CD), and the 300 kDa cation-independent (CI) Mannose 6-phosphate receptors (CD-MPR and CI-MPR, respectively). These receptors are type I transmembrane domain proteins that co-operate in the delivery of lysosomal proteins but also have non-overlapping functions in the targeting of a subset of lysosomal enzymes [53,54] (Figure 1). The MPRs are found in vertebrates including zebrafish (Danio rerio), but it is not well defined in invertebrates or other organisms [55]. Beyond transport of lysosomal proteins, CI-MPR, also known as insulin-like growth factor II receptor (IGFRII), also plays a significant role in the clearance of IGFII hormone through endocytosis [56,57].

The binding of M6P tagged proteins to the receptors is a pH-dependent process: the relatively neutral pH of the TGN (pH 6,5) results in conformational changes of the receptors, enabling their interaction with M6P-tagged cargos. Once delivered in the more acidic environment of the lumen of the endosomal compartment (pH≤5), cargos are dissociated from the receptor [58,59]

In addition to M6P, a study combining NMR spectroscopy and molecular modelling showed that CI-MPR can also bind Man-P-GlcNAc residues via domain 5 of its 5th mannose 6-phosphate receptor homology (MRH) domain. Man-P-GlcNAc containing proteins are cargos that eluded the GlcNAc hydrolysis catalyzed by the uncovering enzyme, exposing the M6P residue [60]. This suggests that cargos which didn’t undergo complete maturation in the Golgi apparatus could still be sorted at the TGN. This could also explain why mice lacking the uncovering enzyme still showed some enzyme sorting to the lysosome [61].

### Sortilin

It has been observed, that despite defects in their MPR trafficking pathway, some cell lines still showed a significant level of lysosomal activity, suggesting that a MPR-independent sorting of lysosomal enzymes exists [62,63]. Sortilin, a type I transmembrane protein, has been identified due to its high homology to yeast Vps10p [64]. Vps10p functions in the sorting of soluble lysosomal proteins between the TGN and endosomes [65]. Sortilin is a cargo receptor that cycles between the TGN and endosomes, and sorts various proteins such as GM_2_AP (GM_2_ activator protein), acid sphingomyelinase and prosaposin, as well as cathepsins D and H to lysosomes [66–68] (Figure 1). Sortilin also localizes to the plasma membrane and regulates the endocytosis of progranulin, lipoprotein lipase, apolipoprotein A-V and EGFR [69–72]. Beyond its role in intracellular trafficking, sortilin was shown to be associated with extracellular vesicles regulating vascular calcification [73].

The cytosolic tail of sortilin is the site of various forms of post-translational modifications (PTMs) regulating its function. Phosphorylation can occur at serine 793 via the p21-activated kinase family [74]. This modification appears to modulate the interaction between sortilin and Adaptor Protein 1 (AP-1), as constitutively active phosphomimetic constructs of sortilin were unable to bind AP-1 in a yeast 2-hybrid system [74]. A second phosphorylation site in the cytosolic tail of sortilin has also been identified. Serine 825 is phosphorylated, and phosphorylation at this site modulates GGA1 binding [75]. Beyond phosphorylation, palmitoylation and ubiquitination are also PTMs found in the cytosolic tail of sortilin. Palmitoylation is required for the efficient endosome-to-TGN retrieval of sortilin, while ubiquitination labels the protein for lysosomal degradation [76,77]. Interestingly, these two PTMs interplay with one another as non-palmitoylated sortilin is ubiquitinated, and degraded in lysosomes [77].

### LIMP-2

LIMP-2 is a type III lysosomal integral protein, which binds β-glucocerebrosidase (β-GC) and traffics it to lysosomes in a pH-dependant manner [78]. Interestingly, the interaction of LIMP-2 with β-GC is suggested to occur in the ER, unlike cargo binding to sortilin and MPRs, which take place in the TGN [78]. Sorting of LIMP-2 to the lysosomal compartment requires its interaction with AP-1 via its cytosolic tail through both dileucine and tyrosine signals [79,80]. More recent studies have shown that LIMP-2 could be more than a lysosomal protein sorting receptor. In fact, LIMP-2 is also involved in the transport of cholesterol and phospholipids such as phosphatidylserine [81,82].

Additionally, the overexpression of LIMP-2 in COS cells results in enlarged early and late endosomes, which is suppressed by dominant-negative expression of Rab5b; a small GTPase that functions as a molecular switch for early to late endosome progression [83]. Lastly, LIMP-2 has been implicated in other neurological diseases such as Parkinson’s disease, Gaucher disease and progressive myoclonic epilepsy (reviewed extensively here [84]), demonstrating its importantance, but partially understood role in lysosomal function.

### Sorting at the TGN: the role of the Clathrin adaptors

Transport of soluble lysosomal proteins from the TGN to the endolysosomal compartment is mediated by clathrin coated vesicles. Their formation, and the specific selection of cargos to sort, is regulated by the cooperation of cargos, cargo receptors, clathrin adaptor proteins, accessory proteins and clathrin, at the interface of the cytosol and the lumen of the trans-Golgi Network. There are two major types of clathrin adaptors: The multimeric adaptor proteins, and the Golgi-localized, γ-ear containing, ADP-ribosylation factor-binding (GGA) proteins.

### Multimeric adaptor protein

**Figure 4:**
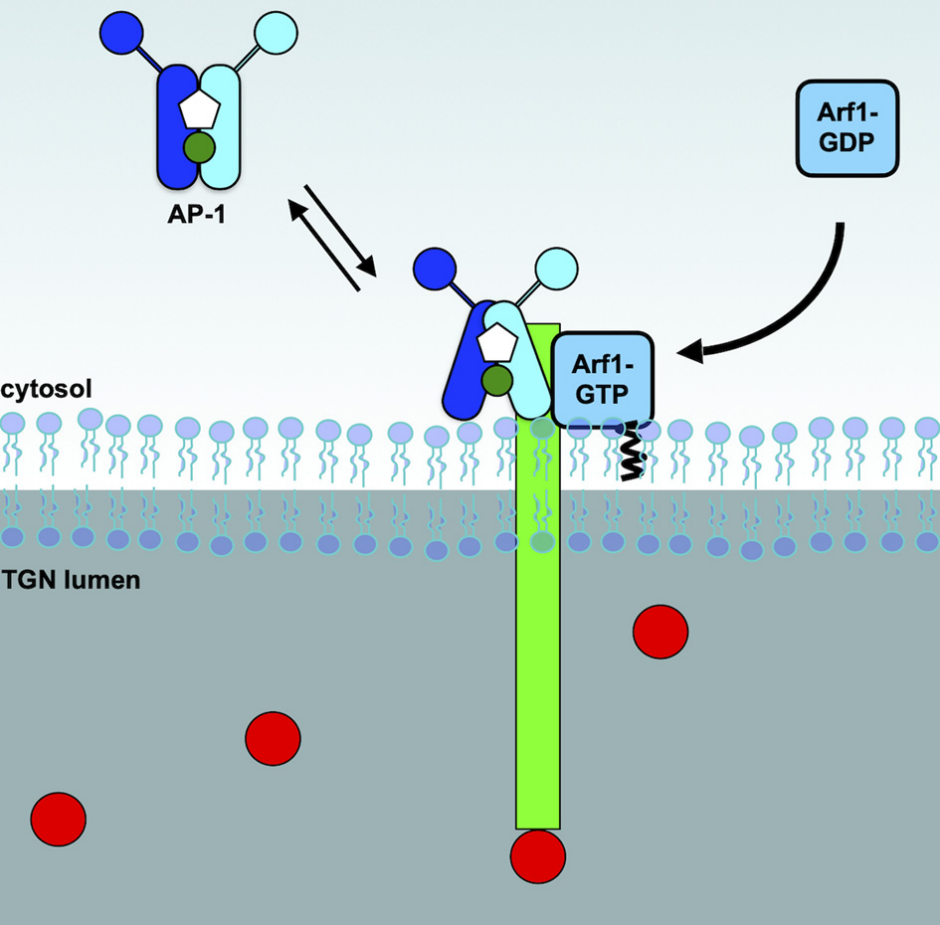
Sorting of the lysosomal sorting receptors at the TGN - AP-1 AP-1 is recruited to the membrane of the TGN by the small GTPase Arf1, which also open the conformation of AP-1. This enables the binding of AP-1 to the sorting receptor (green), which is loaded with soluble lysosomal cargo (red circles).

Adaptor protein-1 (AP-1) is a heterotetrameric protein complex composed of two large subunits (β1 and γ): a medium subunit (µ1) and a small subunit (σ1). The complex is arranged into two sections: a core composed of the N-terminal portions of β1 and γ, and the entire µ1 and σ1 subunits, while the C-terminal portions of β1 and γ extend out away from the core [9]. AP-1 can interact with the cytosolic tail of CD-MPR, CI-MPR and sortilin through specific motifs (Figure 4). Two such motifs are contained in the tail of each receptor, the tyrosine motif (YXXØ, where X is any amino acids and Ø is a bulky hydrophobic amino acid) and the dileucine motif ([D/E]XXXL[L/I]) [85,86]. Binding of the tyrosine motifs to AP-1 is mediated by the µ1 subunit [85,87], while dileucine motifs binding requires σ1 and the core portion of γ [86,88].

Beyond these classical sorting signals, atypical sorting motifs have also been identified for proper lysosomal trafficking. For example CLN3, a multi-spanning membrane protein requires the extended dileucine motif [EEEX(8)LI] in its second (large) cytoplasmic loop domain and the [MX(9)G] motif, where methionine and glycine, separated by nine amino acids in its C-terminal tail, for its proper lysosomal localization [89]. AP-1 has been shown to bind the atypical sorting motifs in CLN3 for its trafficking; however, there are contradictory results whether AP complexes binds these motifs or not [90,91].

The interaction between the sorting receptor and AP-1 occurs on the membranes of the TGN. The spatiotemporal recruitment of AP-1 to membranes requires phosphatidylinositol 4-phosphate (PI4P) and the small GTPase Arf1 [92–94]. Small GTPases such as Arf1 cycle between an inactive cytosolic form which is bound to GDP, and a membrane bound active form [95]. Guanine nucleotide exchange factors (GEFs) activate small GTPases, while GTPase activating proteins (GAPs) shut off the signal, returning small GTPases to the cytosol [96]. Activation of Arf1 for AP-1 recruitment is modulated by BIG2 [97,98], while the GAP implicated in this pathway has not been fully determined. Beyond membrane recruitment, Arf1 activates AP-1 by changing its conformation leading to an increased binding to the cytosolic tail of the sorting receptors [99,100].

Beyond binding to proteins sorted toward the lysosomal compartment, AP-1 has also been implicated in endosome-to-TGN sorting of a wide range of cargos including CI-MPR and CD-MPR [101,102], and TGN-to-PM sorting. AP-1 also plays an important role in basolateral sorting in polarized cells [103] and is essential for embryonic development in mice. Knockout of the γ1 or µ1a subunits of AP-1 in mice results in embryonic lethality [104,105]. Mutations in AP-1 subunits cause a range of human disorders, which are classified as coatopathies [106]. For example, X-linked intellectual disability and Pettigrew syndrome are associated with mutations in the σ1B gene [107,108]. On the other hand, mutations in σ1A gene cause MEDNIK syndrome (**m**ental disability, **e**nteropathy, **d**eafness, **n**europathy, **i**chthyosis and **k**eratoderma), and loss-of-function mutation in the β1 gene results in MEDNIK-like syndrome [109–111]. Mutations in the σ1C subunit of the AP-1 complex lead to pustular psoriasis, an autoinflammatory skin condition [112,113].

MEDNIK syndrome is characterized by several symptoms including profound intellectual impairment, deafness, cerebral motor disorder, severe intestinal impairments and scaly/thickened skin. Mutations in the *AP1S1* gene encoding the σ1A subunit of the AP-1 protein complex have been identified as the cause of MEDNIK syndrome [114,115]. The most common mutation results in the introduction of a premature stop codon at the beginning of exon 4, translating to a truncated protein of 19 amino acids [114].

### The Golgi-localized, γ-ear containing, ADP-ribosylation factor-binding proteins

**Figure 5:**
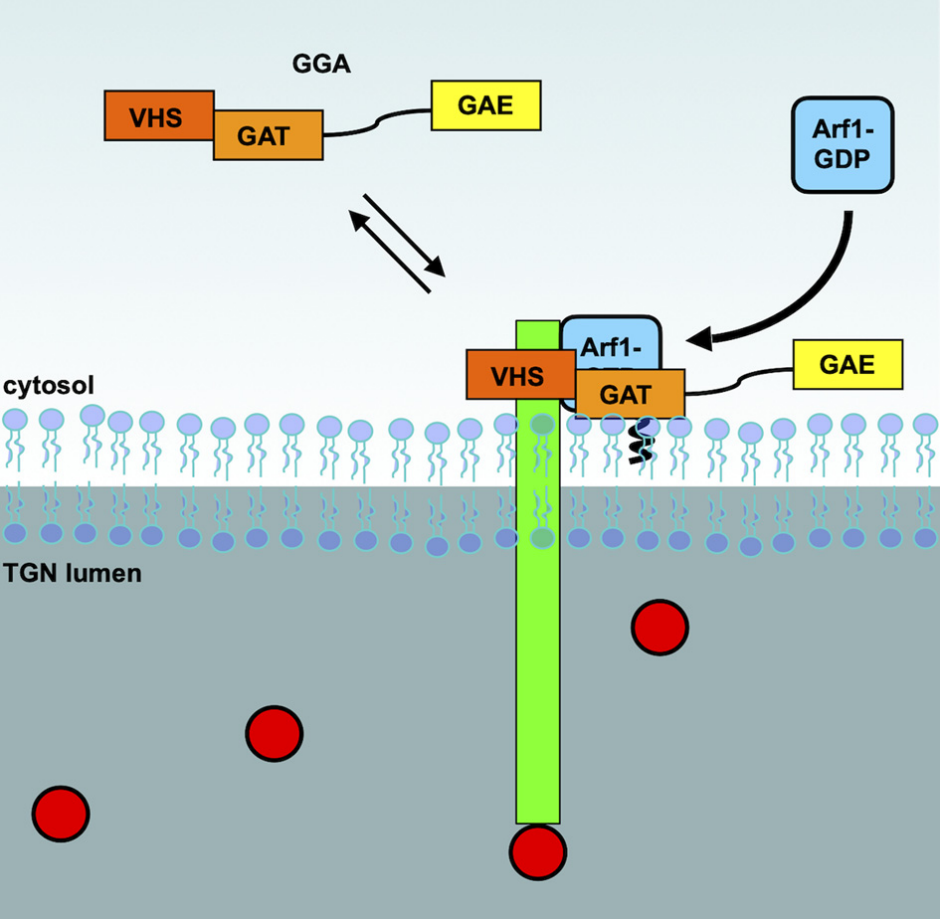
Sorting of the lysosomal sortig receptors at the TGN - GGAs GGA proteins are recruited to the TGN membranes by Arf1, which enables their interaction with cargo (red dots) bound sorting receptor (green).

The GGA (GGA1-3) proteins are monomeric adaptors of 65–80 kDa, which were originally discovered as Arf interacting proteins [116–118]. The GGAs contain four domains; VHS (Vps27p/Hrs/STAM), GAT (GGA and TOM1), hinge and GAE (gamma-adaptin homology domain). As the name implies, the C-terminus of the GGA proteins show high sequence homology with the γ subunit of AP-1 complex [119]. In mammalian cells, the GGA proteins localize mainly to the TGN, and similarly to AP-1, bind to sortilin and the mannose 6-phosphate receptors through the interaction of their dileucine signals with the VHS domain [120–122] (Figure 5). The GAT domain is required for the TGN localization of the GGAs, and is also required for their interaction with Arf1 [116,123]. The hinge domain contains clathrin binding sites, and has been shown to interact with this protein [123,124], while the GAE domain binds several accessory proteins [125,126]. The hinge domain of GGA and the γ ear domain of AP-1 were shown to interact, which suggests that GGA can function along with AP-1 to sequester sorting receptor into AP-1 positive vesicles [127]. In HeLa cells, where all three GGAs were deleted using CRISPR/Cas9 (GGA^KO^), CI-MPR localized mainly to the Golgi, in contrast with wild-type cells where CI-MPR showed both Golgi and peripheral puncta staining. Moreover, in the triple GGA^KO^ cells, cathepsin D localization to endolysosomes was impaired, however not completely abolished, suggesting that AP-1 could maintain its trafficking [128].

GTP-loaded Arf1 is required to recruit GGAs to the TGN [116,123]. However, the mechanism regulating the Arf1 cycle for GGA recruitment is not well understood. The GEF GBF1 has been implicated in the recruitment of GGAs, as its depletion using RNAi resulted in decreased GGA3 recruitment, whereas expressing catalytically dead GBF1 led to the mis-sorting of the soluble lysosomal protein prosaposin [129]. Depletion of the GAP ArfGAP3 caused a decreased membrane recruitment of the GGAs, which resulted in the mis-sorting of the soluble lysosomal enzyme cathepsin D. However, CI-MPR was relocalized to endosomes in ArfGAP3 deleted cells, suggesting a blockage in endosome-to-TGN trafficking [130].

It is not fully understood how GGAs function in relation to AP-1 at the TGN. Some work has suggested that GGAs hand off cargo to AP-1, in order to sort cargo at the TGN. More work will be required to fully understand the role of these proteins in lysosomal sorting.

## Sorting at endosomes

### Cargo receptor retrieval to the TGN

**Figure 6:**
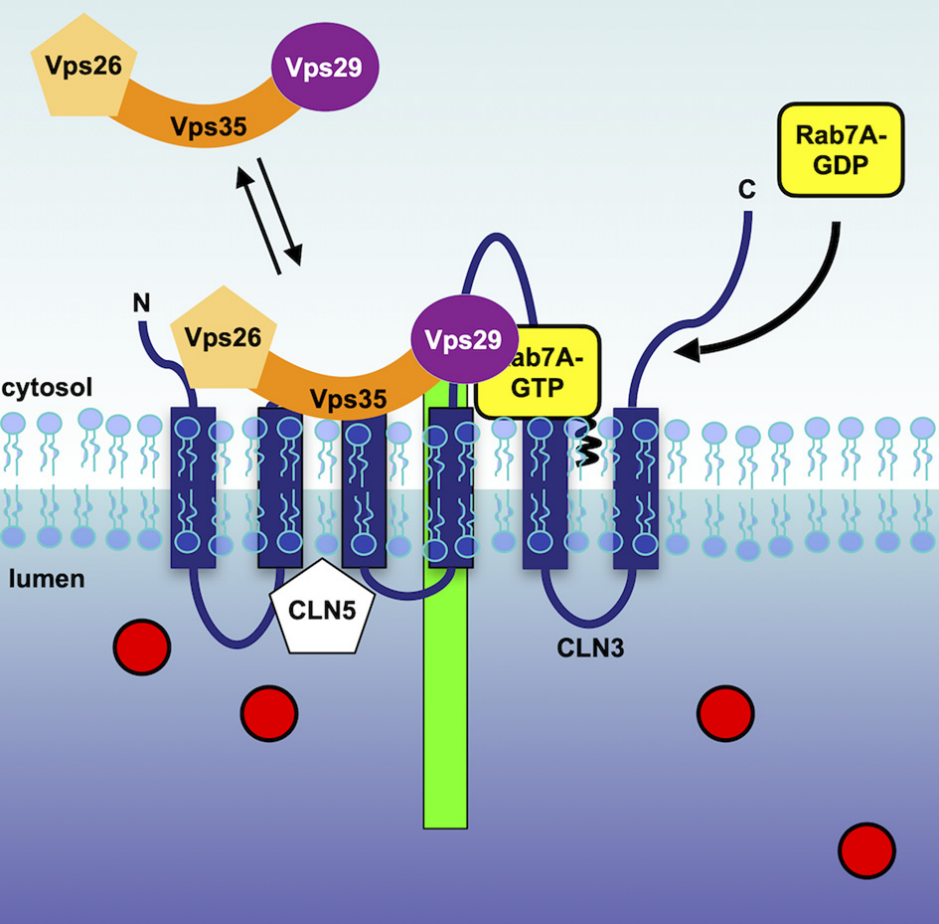
Retrieval of the lysosomal sorting receptors from the endosome In order to retrieve the lysosomal sorting receptors (green) from the endosome, Rab7A is recruited to membranes and enables the binding of retromer to the receptor CLN3 (blue) and CLN5 coordinate this process.

Once the receptor/cargo complex that was packaged into clathrin-coated vesicles and fuses with the late endosome, the more acidic pH in this organelle causes a conformational change in the receptor, which enables cargo to be released. Meanwhile, the receptor is sorted out of the endosome and retrieved to the TGN. The receptor escapes lysosomal degradation and is instead used for subsequent rounds of sorting. Originally identified in yeast, retromer plays a key role in retrograde trafficking and receptor recycling. Retromer is a cytosolic protein complex that is recruited to endosomal membranes and can interact with the cytosolic tail of the receptors (Figure 6). This trimeric complex is composed of vacuolar protein sorting (Vps)-26, Vps29 and Vps35 [131,132]. In retromer-depleted cells, the retrieval of the receptors is less efficient, resulting in their lysosomal degradation (Figure 1). This has a significant negative impact on the sorting of soluble lysosomal cargos, resulting in lysosomal dysfunction [131,132].

The spatiotemporal recruitment of retromer is regulated by a small GTPase, Rab7A (Figure 6). In its GTP active form, Rab7A is recruited to membranes and stabilizes the recruitment of retromer [133,134]. In Rab7A-depleted cells, retromer recruitment is significantly reduced, but the receptors are not degraded, as Rab7A is also required to enable lysosomal degradation. Instead, the receptors accumulate at the late endosome [133].

As a small GTPase, the recruitment of Rab7A to membranes is regulated by its GTP loading status [135]. In an inactive form, Rab7A is bound to GDP and is localized to the cytosol. The GEF MON1/CCZ1 loads Rab7A with GTP, which enables its membrane recruitment [136,137]. Two GAPs, TBC1D5 or TBC1D15, hydrolyze GTP back to GDP, resulting in Rab7A shutoff [134,138,139]. Beyond this mechanism, Rab7A can be regulated specifically in its function of retromer recruitment. First, it is known that several post-translational modifications on Rab7A can regulate its function. Rab7A has been shown to be phosphorylated [140–143], palmitoylated [144] and ubiquitinated [145,146]. Of these, palmitoylation is required for the interaction between Rab7A and retromer, although nonpalmitoylatable Rab7A is still membrane localized and can mediate the degradation of internalized cell surface proteins [144].

Beyond PTMs, other mechanisms have been shown to regulate the retrieval of the lysosomal sorting receptors. For instance CLN3, an integral membrane protein localized to endolysosomal membranes acts as a scaffold to favor the Rab7A/retromer and retromer/sortilin interactions as CLN3 can interact with all three of these proteins [147]. In CLN3-depleted HeLa cells, these interactions are significantly weakened, resulting in the receptor degradation in lysosomes and mis-processing of cathepsin D. Additionally, CLN5, which is a soluble lysosomal protein that binds CLN3, is also required for retromer recruitment, as it modulates CLN3 activity [148]. In CLN5-depleted HeLa cells, the Rab7A/retromer and retromer/sortilin interactions are also disrupted, as are the CLN3 interactions with Rab7A, retromer and sortilin. This also leads to receptor degradation in lysosomes and defective lysosomal function. In this case, CLN5 appears to regulate Rab7A PTMs, as its palmitoylation level is significantly reduced in CLN5-deleted HeLa cells [148]. Although the mechanism of Rab7A palmitoylation is not known, one could speculate that CLN5 modulates an interaction of the palmitoylation machinery with CLN3, promoting Rab7A palmitoylation.

Much like mutations in CLN6 or CLN8, mutations in either CLN3 or CLN5 in humans also cause NCL. No cure has yet been found for these diseases which dramatically reduce the lifespan of patients, with death often occurring before the third decade of life. Beyond the NCLs, retromer mutations have been identified in Parkinson's disease [149–152], while defective retromer has also been associated with Alzheimer’s disease [153,154] and amyotrophic lateral sclerosis (ALS) [155]. Finally, mutations in Rab7A have been shown to cause Charcot–Marie–Tooth disease (CMT), a peripheral nerve disease which induces loss of muscles and sensory defects [156].

Retromer is not the only complex regulating the retrieval of proteins from endolysosomes to the TGN. Indeed, it has been shown that a combination of sorting nexin (SNX) proteins can interact directly with CI-MPR for its recycling either to the TGN or to the plasma membrane [157]. This sorting complex has been termed Endosomal SNX–BAR sorting complex for promoting exit-1 (ESCPE-1) (Figure 1), and is composed of SNX1 (or SNX2) and SNX5 (or SNX6). While SNX5 and SNX6 bind a specific motif in the cytosolic tail of CI-MPR, SNX1 and SNX2 interact with PI3P at the endosomal membrane and initiate the formation of tubulo-vesicular transport carriers, via their C-terminal BAR (Bin/Amphiphysin/Rvs) domain [157].

Two distinct sorting mechanisms function at endosomes to retrieve receptors. Why the cell needs these two mechanisms is unclear. Perhaps the redundancy ensures proper retrieval. What is emerging, is that these two pathways package cargo receptors into distinct vesicles that reach the TGN and tether through different mechanisms [158,159].

### Soluble lysosomal protein delivery to their final destination

As the soluble lysosomal proteins are progressively dissociated from their receptors and the receptors are retrieved back to the TGN, endosomes continue their routes towards lysosomes. Endosomal pH slowly decreases to reach lysosomal pH 4.5, resulting in final cleavage and activation of lysosomal hydrolases such as cathepsin D [160,161]. When in close proximity, late endosome and lysosome membranes tether and can undergo heterotypic fusion, resulting in material exchange between the two compartments. The mechanisms behind endosome/lysosome fusion is still a matter of debate, although two models have been proposed: the ‘kiss and run’ and the ‘fusion-fission’ models. The first is based on transient fusions and fissions events between the two compartments, enabling content mixing [162,163]. On the contrary, the ‘fusion-fission’ model suggests that late endosome and lysosome completely fuse to form a hybrid organelle called endolysosome. Later on, lysosomes would reform, packing lysosomal proteins with it and serving as an enzyme storage compartment, until the next fusion with another organelle [164,165]. Evidence also suggests that both mechanisms could occur concurrently in mammal cells [166].

Fusion events between late-endosomes and lysosomes are a three-step process: membrane tethering, followed by SNARE complex assembly, which results in the twio membranes fusing. It is mediated and regulated by several proteins. In mammalian cells, the homotypic fusion and vacuole protein sorting (HOPS) complex is one of the main actors involved. This tethering complex has a seahorse-like structure and is composed of six subunits : VPS11, VPS16, VPS18, VPS33, VPS39 and VPS41. HOPS bridges membranes from different compartments and keep them in close proximity. In yeast, the Rab7A homolog Ypt7 was showed to be involved in the recruitment of VPS39 and VPS41 to membranes [167,168]. In mammalian cells, the recruitments of those subunits to the endosomal membranes would not be directly mediated by Rab7A, but through its effectors RILP [169], and Pleckstrin homology domain-containing family M member 1 (PLEKHM1) [170]. However, Rab7A could play an indirect role in endosome fusion, through the formation of a complex with RILP and the cholesterol sensor ORP1L. Rab7A–RILP–ORP1L complex would regulate the coordination of endosome movement and fusion, and the switch from the transport machinery to the fusion machinery [169,171]. The activated small GTPase Arl8 is also responsible for the recruitment of VPS41 to the lysosomal membrane [172,173]. In addition, Arl8 and Rab7 were also shown to collaborate to recruit PLEKHM1 onto lysosome and late endosome membranes respectively [174]. PLEKHM1 is an essential adaptor protein which promotes the recruitment and stabilization of HOPS subunits, and the tethering of endosome and autophagosome membranes with lysosomes [170]. HOPS complex assembles and enables the recruitment of SNARE proteins [175]; for example, a study conducted in yeast found that VPS33 is the subunit where the interaction with SNARE proteins occurs [176,177].

In mammalian cells, trans-SNARE complexes consist in the association of one R-SNARE (VAMP7 or VAMP8) in association with three Q-SNAREs (Syntaxin-7, Syntaxin-8 and VTI1b). VAMP8 would be involved in homotypic fusion of endosomes whereas VAMP7 would be required for heterotypic fusion of endosomes with lysosomes [178]. SNARE association acts as a zipper that brings membranes close enough to each other to result in membrane curvature change and spontaneous proteolipid fusion pore formation [179]. Lumen content of both organelle then mix, leading to soluble enzyme release in endolysosomes.

## Conclusions

Sorting of soluble lysosomal proteins is a fundamental cellular process required to maintain lysosomal activity. It involves the close cooperation of a wide range of proteins from various compartments, which act together to ensure the proper localization and maturation of soluble lysosomal proteins. The variety of protein adaptors, cargo receptors and recognized sorting motifs enable the sorting numerous proteins with high specificity. For a number of years, defects in this process have been known to cause lysosomal storage disorders [1], but it is only more recently that they have been associated to more common human diseases, especially age-related neurodegenerative diseases [153–155,180]. Those studies have especially highlighted the underestimated importance of the retrograde trafficking of receptors and misrouted proteins, in the process and the regulation of lysosomal protein sorting. Although some of those pathways have been investigated for more than 40 years, mechanisms regulating the sorting of lysosomal protein have not been fully elucidated. Revisiting models and identifying new effector functions, using novel methods such as protein-protein interactions studies in live cells, CRISPR/Cas9 knockouts generation, and unbiased ‘omics’ approaches, appear to be essential for a better understanding of the sorting of lysosomal proteins. Increasing our comprehension of these pathways could hence lead to the identification of novel therapeutic targets for a number of diseases.

## Abbreviations

β-GC: β-glucocerebrosidase
CMT: Charcot–Marie–Tooth disease
GAE: gamma-adaptin homology domain
GAP: GTPase activating protein
GEF: guanine nucleotide exchange factor
HOPS: homotypic fusion and vacuole protein sorting
PLEKHM1: Pleckstrin homology domain-containing family M member 1
